# Clinical study on the establishment of radio-cephalic autogenous arteriovenous fistulas in small blood vessels by multi-segment balloon dilation technique

**DOI:** 10.1097/MD.0000000000039758

**Published:** 2024-09-20

**Authors:** Qiyu Kang, Yajie Hao, Huifeng Zhang, Weimin Yu, Xiaoguang Huang

**Affiliations:** a Department of Nephrology, Shanxi Bethune Hospital, Shanxi Academy of Medical Sciences, Third Hospital of Shanxi Medical University, Tongji Shanxi Hospital, Taiyuan, China.

**Keywords:** arteriovenous fistulas, balloon angioplasty, multi-segment balloon, patency, small vessels

## Abstract

**Background:**

To investigate the effect of dilating small blood vessels using a balloon dilation (BD) technique on the occurrence of radio-cephalic autogenous arteriovenous fistulas in terms of patency, blood flow, and vein diameter (VD).

**Methods:**

The subjects included in this study were all patients with chronic renal failure and required radio-cephalic arteriovenous fistula surgery for the first time and had not received dialysis before. Patients with VDs <2 mm were included as study subjects. They were either assigned treatment using a BD group or a control group that received hydrostatic dilation. The differences between the 2 groups were analyzed in terms of patency, blood flow, and VD.

**Results:**

A total of 22 patients were enrolled in the balloon dilatation group and 20 patients in the control group. The diameters of cephalic veins (mm) of the experimental and control group were compared at various time points: immediately postoperation, 2.89 ± 0.42 versus 1.99 ± 0.28 (*P* < .001); 1 week later, 3.16 ± 0.59 versus 2.66 ± 0.60 (*P* = .022); 1 month later, 3.76 ± 0.91 versus 3.18 ± 0.83 (*P* = .087); and 2 months later, 4.08 ± 1.15 versus 3.38 ± 1.13 (*P* = .169). Brachial artery flows (mL/min) of the 2 groups at various time points were given as follows: immediately postoperation, 413.49 ± 145.09 versus 235.61 ± 87.77 (*P* < .001); 1 week later, 563.26 ± 206.83 versus 331.30 ± 126.78 (*P* < .001); 1 month later, 679.34 ± 218.56 versus 376.79 ± 156.25 (*P* < .001); and 2 months later, 736.31 ± 202.61 versus 394.60 ± 161.96 (*P* < .001). The primary patency at 1 year for the experimental group was 61.9% compared to 11.1% for the control group (*P* = .045). Similarly, the secondary patency rates at 1 year were 90.5% for the experimental group and 55.6% for the control group (*P* = .030). The results showed that the functional primary patency rate within 1 year was 57.1% versus 16.7% (*P* = .032), and the functional secondary patency rate within 1 year was 85.7% versus 50.0% (*P* = .038).

**Conclusion subsections:**

BD has obvious advantages over hydrostatic dilation for chronic renal failure patients with small veins in establishing arteriovenous fistula in terms of patency and blood flow.

## 1. Introduction

Arteriovenous fistulas were first proposed in 1966.^[1]^ Since then, vascular access has been further developed, including the introduction of autogenous arteriovenous fistula (AVF), arteriovenous graft,^[2]^ and percutaneous arteriovenous fistulas.^[3]^ AVF is still the first choice^[2]^ although there is the risk of immature and thrombosis, which can lead to the failure of the fistula and affect the survival of patients with end-stage renal failure.

The failure of AVF is related to factors such as age, gender, hypertension, diabetes, peripheral vascular disease, and absence of surgical experience.^[4–7]^ Among these factors, the diameter of the anastomotic vein plays an important role in the failure. In practical clinical work, we will encounter many patients with small blood vessels in the forearm. Because of the difficulty of autologous arteriovenous fistula of small blood vessels, it poses a great challenge to the vast majority of surgeons. For the radio-cephalic AVF, the guidelines recommend that the minimum artery diameter ≥1.5 mm and the vein diameter (VD) ≥2 mm for the first autologous arteriovenous fistula^[2]^ (after the bundle arm). Mendes et al^[8]^ have reported that in left forearm AVF, when the diameter of the cephalic vein is>2 mm, the maturity rate of AVF can reach 76%. When the diameter of the cephalic vein was <2 mm, the maturity rate of AVF was reduced to 16%. It can be seen that the immature rate and failure rate of small vascular fistula are higher than those of normal vascular fistula. The patency and maturity rates of AVFs established in small intravenous fistulas (<2 mm) can be improved by a variety of methods, such as liquidity dilations, and enlarging the anastomotic diameter. Therefore, our study aims to compare the differences between balloon dilation (BD) and hydrostatic dilation (HDs) in terms of AVF patency, blood flow, and VD after establishing autologous arteriovenous fistula in order to explore the optimal surgical approach.

## 2. Materials and methods

### 2.1. Patient recruitment

All subjects were patients with chronic renal failure who underwent radiation-cephalic arteriovenous fistula surgery for the first time and had never received dialysis before. Estimated glomerular filtration rate of ≤15 mL/(min/1.73 m^2^). The patients were recruited from the Department of Nephrology, from August 1, 2018, to September 1, 2021. Those patients with carpal cephalic VD <2 mm were selected as the subjects of the study. All patients signed informed consent forms and obtained support from the Ethics Committee of Shanxi Bethune Hospital. In addition, arterial and vascular calcification, thrombosis, and other lesions affecting vascular elasticity were excluded in order to ensure venous elasticity. Depending upon the technique used for AVF establishment, these patients were randomly divided into an experimental group (BD) and a control group (HD). Data were collected for the patency rate, blood flow in the brachial artery, and the diameter of the vein proximal to anastomosis.

### 2.2. Procedure

#### 2.2.1. Preoperative evaluation

Patients were evaluated before surgery, and informed consent was obtained. The preoperative examination included height, weight, blood pressure, blood coagulation, preoperative immunity, electrocardiogram, cardiac ultrasound, and an Allen test. The vascular course of the upper limb was evaluated by Doppler ultrasound. The operation site was marked, and the preoperative arteriovenous diameter and brachial artery flow (BAF) were measured. The tourniquet was tied 5 cm above the elbow. Venous distensibility was indicated by the difference in diameter of the carpal cephalic vein before and after the tourniquet test. The mean venous distensibility was 0.15 mm (range, 0.12–0.19 mm) in the BD group and 0.14 mm (range, 0.10–0.19 mm) in the HD group (*P* = .516).

#### 2.2.2. Surgical methods

**Figure 1.**
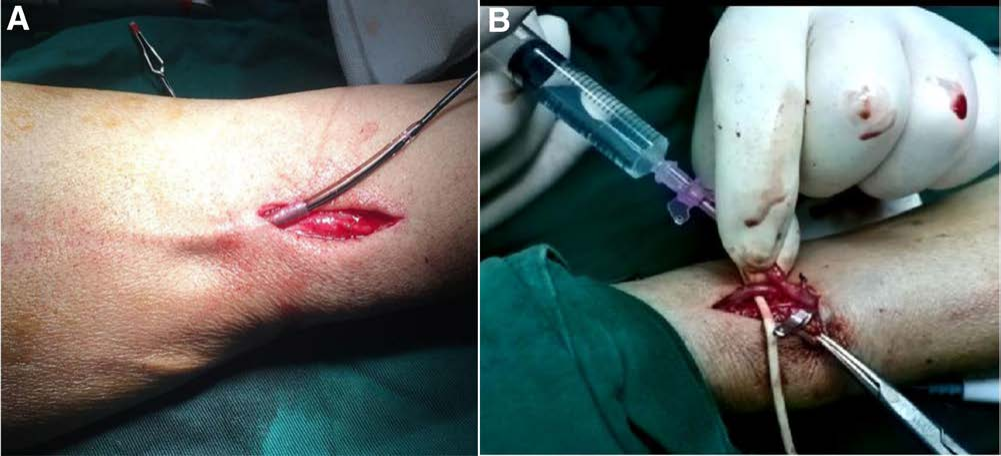
(A) Dilation of a long segment of small veins (<2 mm) by balloon. (B) Dilation of small veins (<2 mm) by injection of heparin saline.

All patients included in this study were treated with radio-cephalic AVF creation. After the preoperative indexes met the surgical indications,^[2]^ the patient was taken to the operating room for brachial plexus anesthesia, routine disinfection, sheet laying, and connection to an oxygen inhalation device and monitor. The surgeon cut the skin at the marked part before the operation, separated the cephalic vein and radial artery, electrocoagulated its prepared small branch vessels, dissociated the 2- to 3-cm radial artery and cephalic vein, and ligated for surgery. The radial artery was cut longitudinally, the cephalic vein was cut obliquely, and the balloon guide wire was inserted along the cephalic vein to the proximal end under ultrasound guidance. After inserting the guide wire, a balloon with a diameter of 3 mm and a length of 15 cm (Produced by Boston Technology) was inserted along the guide wire and connected to a pressure pump. The cephalic vein was dilated under 6- to 10-atm pressure (depending on the patient’s specific condition) for 30 seconds, and multi-segment dilatation was performed up to 15 cm above the elbow. After dilatation, the balloon and guide wire were removed, and the cephalic vein and radial artery were joined using end-to-side anastomosis. Subsequently, the subcutaneous tissue and skin were anastomosed, and the operation was completed. In the control group, the cephalic vein and radial artery were separated and ligated after local anesthesia; the cephalic vein was also cut off obliquely. Heparin saline (20 mL) was injected into the cannula in order to the dilate cephalic vein. The cut end of the vein was pinched by hand for 30 seconds to prevent leakage. The remainder of the procedure was the same as the BD group. The different surgical methods are shown in Figure 1.

#### 2.2.3. Follow-up

Each patient was followed up for 1 year. We measured and recorded the inner diameter of the proximal cephalic vein and the brachial artery blood flow before, after, and 1 week, 1 month, and 2 months after the operation by color Doppler ultrasound, calculated the patency rate and other indicators, and analyzed the differences between the indicators. The patients were then followed up for 1 year after the procedure. An immediately successful operation was defined as finding the palpable and obvious thrill of the AVF vessels and filling of AVF flow under ultrasound. The maturity of the AVF was defined as having a BAF ≥500 mL/min and a diameter ≥5 mm in the outflow tract of the internal fistula.^[9]^

#### 2.2.4. Related definition

Primary patency refers to the time from the establishment of vascular access to the occurrence of complications such as stenosis, occlusion, or thrombosis, during which no surgical intervention to maintain vascular access patency is carried out. Secondary patency refers to the period from the establishment of vascular access to the abandonment of access, during which ≥1 surgical interventions are carried out to maintain the patency of access. The surgical intervention can be due to any reason affecting the patency of access, such as stenosis, occlusion, or thrombosis.^[10]^ Functional primary patency refers to the period from the creation of an arteriovenous fistula (AVF) and its first successful use for hemodialysis, up until the first instance where any form of intervention (whether surgical or interventional radiology) is required to maintain or improve its function. Simply put, it measures the duration an AVF remains usable for dialysis without any interventions. Functional secondary patency, on the other hand, refers to the period from the first successful use of an AVF for hemodialysis, through ≥1 intervention, until the AVF can no longer be used for dialysis. This includes the cumulative time following all interventions carried out to maintain the functionality of the AVF.^[11]^

#### 2.2.5. Statistical methods

IBM SPSS 27.0 was used for statistical analysis. The mean ± standard deviation was used for measurement variables, and the frequency and percentage were used for counting variables. Variables in age, body mass index, cephalic vein internal diameter, and BAF were shown to be in accordance with a normal distribution using the Kolmogorov-Smirnov method. Therefore, the differences between the above variables were tested by an independent sample *t* test. The remaining frequency data were analyzed using the Pearson χ^2^ test. Kaplan–Meier was used to describe the patency of the 2 groups, and Breslow was used to test the difference analysis. The Cox risk ratio model was used for multivariate survival analysis, and the risk ratio and its 95% confidence interval were calculated. The difference was statistically significant when the test level was α = 0.05 (*P* < .05).

## 3. Results

### 3.1. Clinical data comparison

A total of 42 patients were enrolled, with 22 in the balloon dilatation group and 20 in the liquid dilatation group. The baseline characteristics of patients in each group are shown in Table 1.

**Table 1:** Baseline characteristics of the 2 groups.

Variable	Treatment			
	Overall (n = 42)	BD (n = 22)	HD (n = 20)	*P*
Sex, male	18 (45%)	10 (45%)	8 (40%)	.721
Age, yr	58.7 ± 14.2	58.2 ± 15.6	59.3 ± 12.9	.811
BMI, kg/m^2^	23.14 ± 3.58	23.55 ± 3.68	22.78 ± 3.56	.564
Diabetes	22 (52%)	12 (54%)	10 (50%)	.768
Hypertension	29 (69%)	16 (72%)	13 (65%)	.588
Smoke	15 (35%)	9 (40%)	6 (30%)	.461
Preoperative vein diameter, mm	1.45 ± 0.15	1.41 ± 0.16	1.49 ± 0.13	.100
Brachial artery flow, mL/min	38.53 ± 21.71	36.39 ± 19.14	40.65 ± 24.73	.567
Radial artery diameter (<2 mm)	5 (12%)	3 (13%)	2 (10%)	

BD = balloon dilation, BMI = body mass index, HD = hydrostatic dilation.

The basic clinical data of the patients are presented in Table 1. As shown in Table 1, there were no significant differences in basic characteristics, preoperative VD, or BAF between the 2 patient groups. In the study, the average venous distensibility in the BD group was observed to be 0.15 mm, with a variability ranging from 0.12 to 0.19 mm. Conversely, the HD group demonstrated a mean venous distensibility of 0.14 mm, encompassing a range from 0.10 to 0.19 mm. Statistical analysis indicated no significant difference between the 2 groups (*P* = .516).

### 3.2. Clinical data

#### 3.2.1. Fistula data

The AVF operations were all performed by a nephrologist with decades of experience to ensure comparability and reference. Success rates of the surgery in the experimental and control groups were 95.5% and 90%, respectively. The failure cases were due to a thrombus in the internal fistula during the operation so that the obvious AVF thrill could not be touched, and ultrasound showed that there was no blood flow, or decreased blood flow, in the AVF.

**Figure 2.**
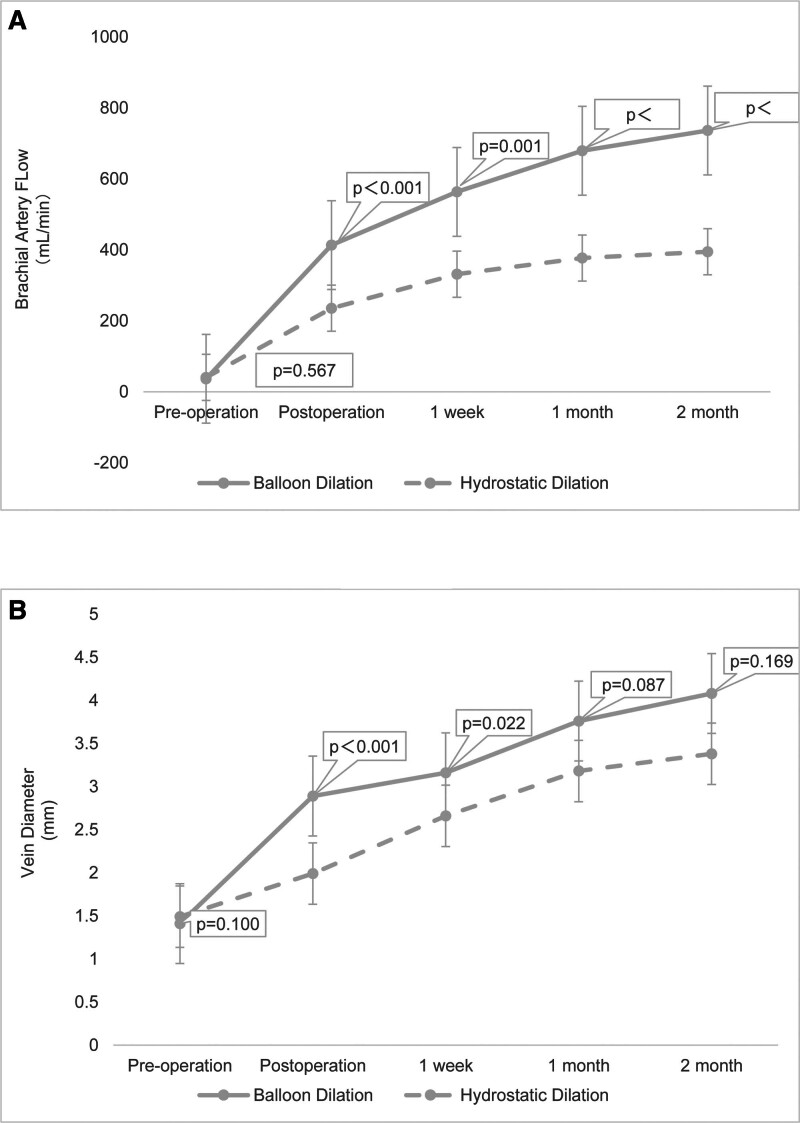
The graph shows (A) brachial artery flow and (B) vein diameter at various time points before and after surgery. The *P* values represent the statistical differences in brachial artery flow and vein diameter between the balloon dilation group and the hydrostatic dilation group at various time points.

The differences in the internal diameter of the cephalic vein and BAF between the 2 groups at various time points are shown in Table 2. With the mean as the ordinate, the trend of the 2 was detailed in Figure 2. Differences in presurgery and postsurgery internal diameters of cephalic veins (mm) and BAF (mL/min) of the experimental and control groups were 1.45 ± 0.41 versus 0.51 ± 0.24 (*P* < .001) and 376.13 ± 141.33 versus 195.81 ± 77.63 (*P* < .001), respectively. The difference was statistically significant.

**Table 2:** Fistula data including vein diameter and brachial artery flow between the 2 groups at various time points.

Variable	Treatment			
	Overall (n = 42)	BD (n = 22)	HD (n = 20)	*P*
Immediately after operation				
VD, mm	2.50 ± 0.57	2.89 ± 0.42	1.99 ± 0.28	<.001
BAF, mL/min	336.57 ± 151.82	413.49 ± 145.09	235.61 ± 87.77	<.001
Preoperative and postoperative differences				
VD, mm	1.04 ± 0.58	1.45 ± 0.41	0.51 ± 0.24	<.001
BAF, mL/min	298.15 ± 147.68	376.13 ± 141.33	195.81 ± 77.63	<.001
1 week after operation				
VD, mm	2.94 ± 0.64	3.16 ± 0.59	2.66 ± 0.60	.022
BAF, mL/min	463.84 ± 209.97	563.26 ± 206.83	331.30 ± 126.78	.001
1 month after operation				
VD, mm	3.53 ± 0.91	3.76 ± 0.91	3.18 ± 0.83	.087
BAF, mL/min	562.29 ± 245.00	679.34 ± 218.56	376.97 ± 156.25	<.001
2 months after operation				
VD, mm	3.83 ± 1.17	4.08 ± 1.15	3.38 ± 1.13	.169
BAF, mL/min	613.29 ± 249.85	736.31 ± 202.61	394.60 ± 161.96	<.001

BAF = brachial artery flow, BD = balloon dilation, HD = hydrostatic dilation, VD = vein diameter.

#### 3.2.2. Fistula maturation time and patency

**Figure 3.**
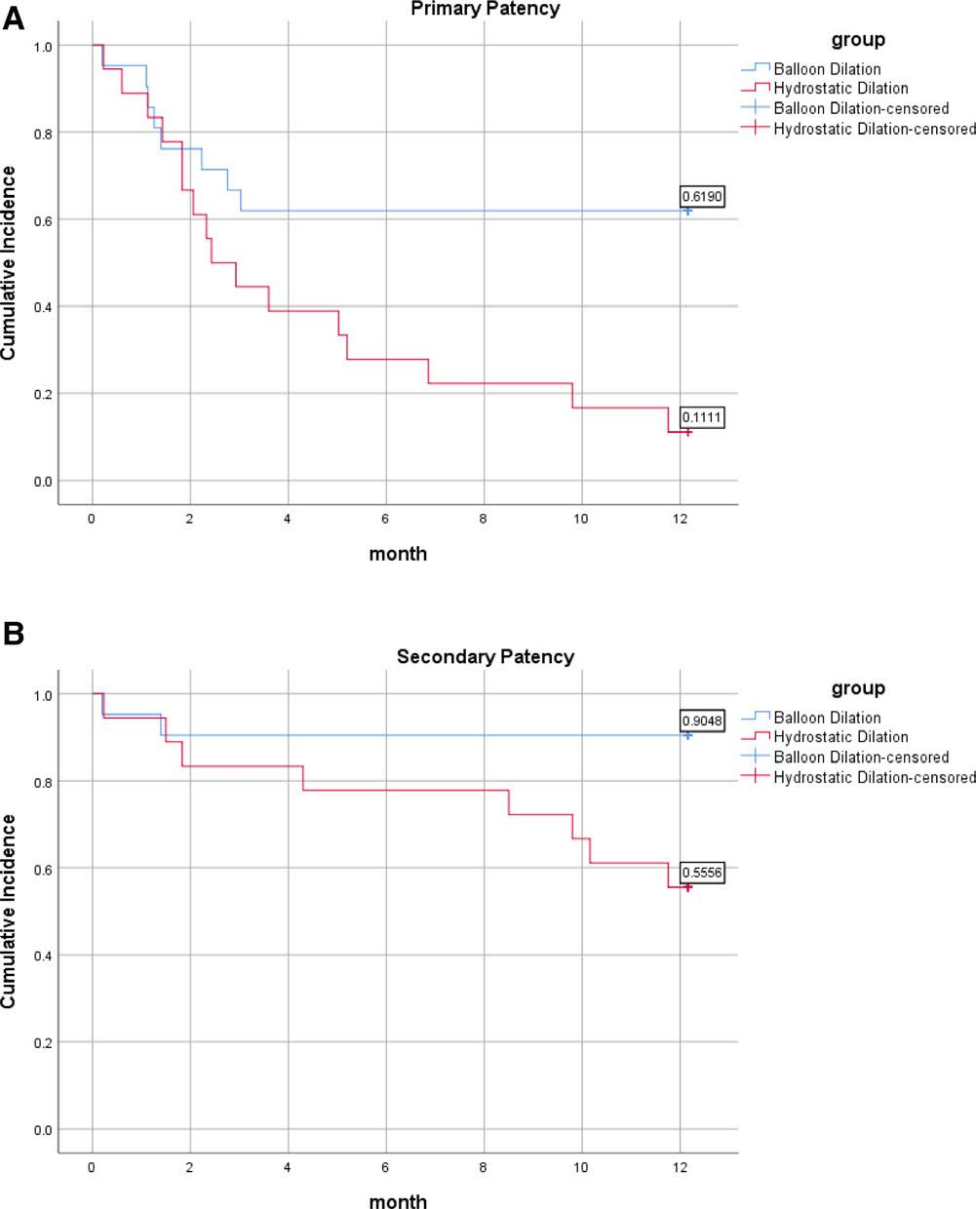
Cumulative incidence plots of vascular access patency rates by procedure type. (A) Primary patency. (B) Secondary patency.

**Figure 4.**
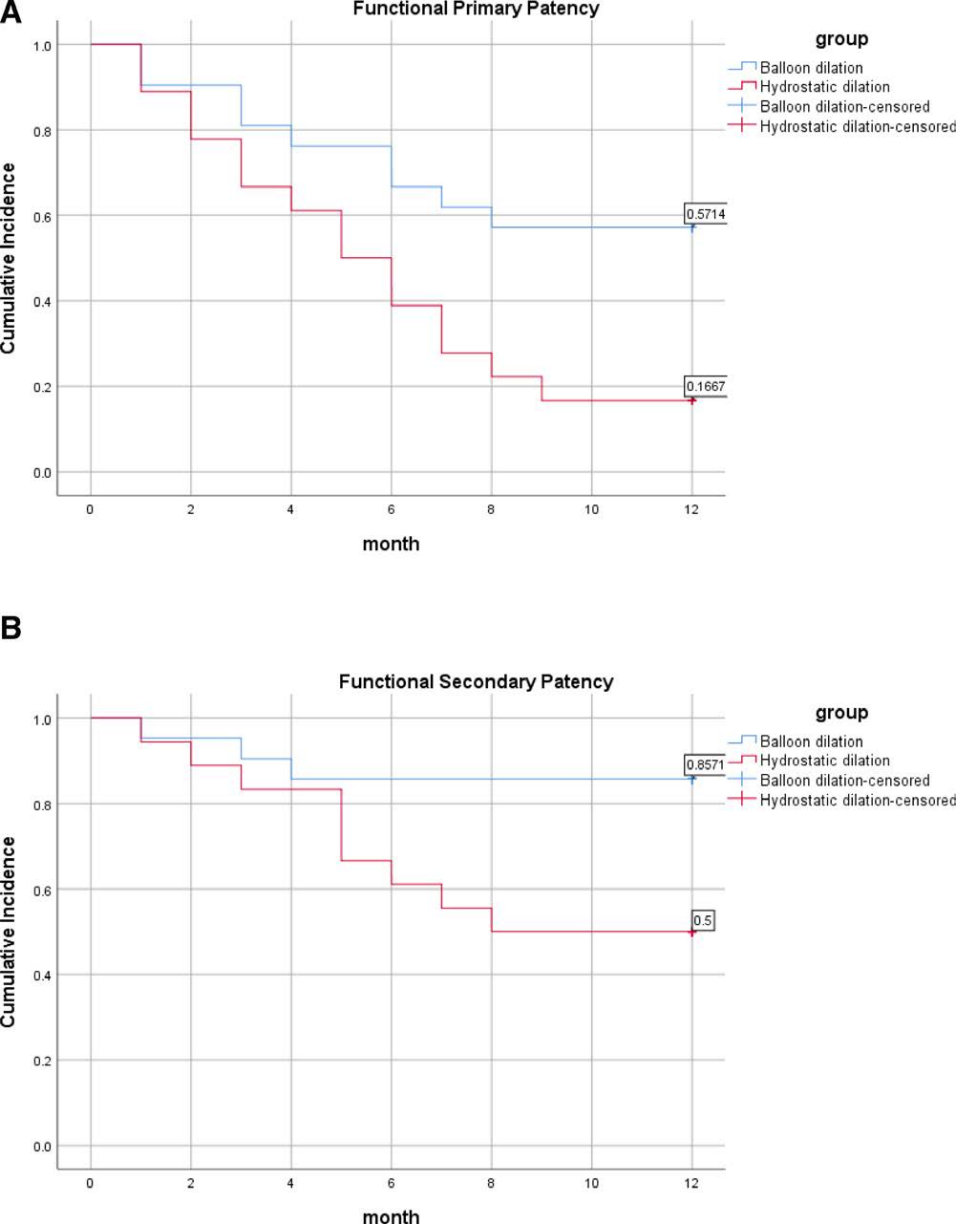
Cumulative incidence plots of vascular access patency rates by procedure type. (A) Functional primary patency rate. (B) Functional secondary patency rate.

Excluding surgical failure cases, the mean time for fistula maturation was 34 (range, 20–44) days in the BD group and 54 (range, 40–73) days in the HD group (*P* < .001). Excluding cases of surgical failure, we observed complete maturation in the BD group, whereas the HD group exhibited 4 instances of nonmaturation. The rates of nonmaturation between the 2 groups were 0% versus 22.22%, respectively (*P* = .089). The primary patency and the secondary patency within 1 year were described by Kaplan–Meier (those patients with failed operations did not participate in the patency analysis). The difference analysis was analyzed using the Breslow test. After calculation, the 1-year primary patency of the experimental group versus the control group was 61.9% versus 11.1% (*P* = .045). The 1-year secondary patency of the experimental group versus the control group was 90.5% versus 55.6% (*P* = .030). The survival curves of primary patency and secondary patency are shown in Figure 3. The survival analysis curve of functional primary patency rate and functional secondary patency rate within 1 year is shown in Figure 4. The results showed that the functional primary patency rate within 1 year was 57.1% versus 16.7% (*P* = .032), and the functional secondary patency rate within 1 year was 85.7% versus 50.0% (*P* = .038).

#### 3.2.3. Cox proportional hazard model

In our study, we further used Cox regression analysis to investigate the effects of balloon dilatation technique, liquid dilatation technique, radial artery diameter, preoperative venous diameter, preoperative brachial artery blood flow, and ipsilateral central venous catheter history on the initial patency and secondary patency of AVF (Tables 3 and 4). The results showed that the balloon dilatation technique was statistically significant in both primary patency and secondary patency (*P* = .038; *P* = .026). In the secondary patency, only the preoperative venous diameter had a statistically significant effect on the patency rate of AVF (*P* = .43), and the hazard ratio was 0.023 (95% confidence interval, 0.010–0.893). This indicates that a larger preoperative VD significantly reduces the risk of AVF failure. Despite the common clinical belief that radial artery diameter, ipsilateral central venous catheter history, and preoperative BAF are crucial factors influencing AVF patency, they did not reach statistical significance in our analysis. This discrepancy could be due to the potential overshadowing effect of unconsidered variables or the sample size being insufficient to detect their effects. Nonetheless, our findings illuminate the complex interplay of complications and vascular anatomy on AVF patency rates.

**Table 3:** Clinical factors associated with primary patency.

Variable	Hazard ratio	95% confidence interval	*P*
Balloon dilation	0.028	0.009–0.805	.038
hydrostatic dilation	0.326	0.168–9.233	.079
Radial artery diameter, mm	0.813	0.217–5.489	.391
Preoperative vein diameter, mm	1.298	0.355–6.953	.410
Preoperative brachial artery flow, mL/min	0.713	0.654–2.328	.384
Ipsilateral CVC history (yes vs no)	1.006	0.102–18.293	.446

CVC = central venous catheter.

**Table 4:** Clinical factors associated with secondary patency.

Variable	Hazard ratio	95% confidence interval	*P*
Balloon dilation	0.016	0.011–0.604	.026
hydrostatic dilation	1.587	1.244–1.989	.349
Radial artery diameter, mm	1.487	1.105–1.763	.296
Preoperative vein diameter, mm	0.023	0.001–0.893	.043
Preoperative brachial artery flow, mL/min	0.997	0.987–1.006	.484
Ipsilateral CVC history (yes vs no)	2.006	0.168–23.893	.582

CVC = central venous catheter.

## 4. Discussion

The ideal procedure for establishing AVF in small blood vessels has been a cause for concern for clinicians since the slender blood vessels are at higher risk of stenosis, plus the risk of endothelial hyperplasia, and immaturity is greater than in ordinary internal fistulas. It has been reported that in the left forearm AVF, the fistula maturity rate can reach 76% when the cephalic VD is >2 mm, but the fistula maturity rate is only 16% when the cephalic VD is <2 mm.^[8]^ Siddiqui et al^[12]^ also demonstrated that the maturity rate of veins with a diameter ≥2.5 mm is 5× higher than that of those with a diameter <2.5 mm. Brimble et al^[13]^ claimed that the cutoff value is 2.6 mm. In those patients with small blood vessels, an arteriovenous graft or a permanent catheter is needed for dialysis treatment if AVF cannot be established. However, this operation is never the first choice for patients with small blood vessels as it has a high probability of infection.

With the development of AVF technology, HD of small veins has become the solution to this problem. HD aimed to expand small VD in order to increase the blood flow and reduce the occurrence of AVF stenosis. However, HD can also present insufficient dilatation of AVF, especially in those patients with poor vascular conditions, or those that are complicated by diabetes or vascular calcification. According to the actual case analysis of HD in our hospital, there was only 11.1% of primary patency and 55.6% of secondary patency in 1 year. Most of the patients underwent percutaneous transluminal angioplasty (PTA) again because of AVF stenosis and insufficient flow, and some of them even abandoned AVF directly and turned to semipermanent or permanent catheterization for maintenance dialysis. According to the statistics from our hospital, some patients can undergo PTA >4 times a year, and the cost of repeated PTA is also a huge expense for patients.

Clinical studies have shown that there are 2 reasons for insufficient HD. First, the dilation pressure is too small. Clinical studies have revealed that the maximum HD pressure was 5 atm, far less than the pressure the BD produced. For patients with vascular calcification and atherosclerosis, HD pressure can be very low. Second, the expansion site is too limited. HD can only dilate a very limited part of the blood vessel from the cut end to the proximal end. This only solves the issue of stenosis at the cut end, but the small blood vessels in the middle and upper parts of the forearm are not dilated, and the high resistance caused by small blood vessels still exists.

The adoption of multi-segment BD of small blood vessels makes up for the shortcomings of the above 2 points related to HD. First, maximum BD pressure (up to 30 atm) is much larger than HD. Indeed, the venous dilatation data for patients in the experimental and control groups were 1.45 ± 0.41 and 0.51 ± 0.42 mm, respectively. In addition, the maximum expansion values of the experimental and control groups were 2.5 and 0.9 mm, respectively. Likewise, we calculated the difference in BAF before and after surgery. The average and maximum increases in BAF of the experimental group were 376.13 ± 141.33 and 677 mL/min, respectively; the average and maximum increases in BAF of the control group were 198.81 ± 77.63 and 309 mL/min, respectively. The 1-year primary patency and secondary patency rates in the balloon dilatation group were 61.9% and 90.5%, respectively, which were significantly higher than those in the hydrostatic dilatation group (11.1% and 55.6%, respectively). At the same time, the functional primary patency rate and the functional secondary patency rate in the balloon dilatation group were 57.1% and 85.7%, respectively, which were also higher than those in the control group (16.7% and 85.7%, respectively). It can be seen that balloon dilatation has incomparable advantages over liquid dilatation in terms of vascular diameter, blood flow increment, and internal fistula patency rate. Clinical observations indicate that adequate blood flow is conducive to the maturity of AVF and can reduce the occurrence of stenosis. Secondly, small blood vessels are progressively dilated from the cut side of the vein using the balloon, up to 15 cm above the elbow. De Marco Garcia et al^[14]^ developed AVF for patients with cephalic VD <3 mm via BD of the cephalic vein. Nevertheless, only a small segment of blood vessels, confined from the wrist to the mid-forearm, was dilated. Thus, many patients developed AVF stenosis very soon after surgery, thereby requiring PTA surgery. Later, Veroux et al^[15]^ improved the Garcia method by performing multi-segment BD on a longer segment of the cephalic vein, from the wrist to the elbow, before creating the AVF. This technique addressed the issue of high resistance in small veins at the proximal end, resulting in a remarkable 95% primary patency and 100% working AVF rate for the AVF established in small veins after 6 months. Building on Veroux et al’s work, we expanded the dilation of small veins to 15 cm above the elbow. In addition to analyzing the patency rates reported by Veroux et al for 6 months, we analyzed patency rates for a longer period of 1 year and the level of secondary patency, which also showed impressive results. The multi-segment dilation used in this study can ensure that all of the small blood vessels dilate more fully, reduce the local resistance caused by vascular slenderness, and, thus, increase the blood flow of AVF. Recently, Sattari et al^[16]^ performed a systematic review and meta-analysis comparing primary balloon angioplasty (PBA) and HD for creating arteriovenous fistulas (AVFs) in end stage renal disease patients’ small-caliber cephalic veins. They assessed 6 dimensions, including primary patency, reintervention, working AVF, immediate success, AVF maturation time, and surgical site infection. Based on their analysis of 3 eligible studies, they found that PBA performed better than HD in all 6 dimensions, which is similar to our research findings. However, Sattari et al’s meta-analysis suffered from small sample size and a lack of long-term follow-up studies. Compared to the 6-month follow-up period in Sattari et al’s meta-analysis, we conducted a longer follow-up period of 1 year, which is a better supplement to Sattari et al’s study. In addition, our patient sample enriched the sample size of the meta-analysis and provided further evidence for the superiority of PBA over HD. In practical clinical work, patients have also been encountered with radial arteries that have an internal diameter of <2 mm. Schild et al^[17]^ suggested that the artery diameter should be >2 mm. For these patients, the method of multi-segment BD was adopted to establish AVF, and all obtained good long-term patency.

However, in this study, balloon dilatation for patients also has the following limitations. First, patients with slender blood vessels often have vascular sclerosis and vascular calcification. Balloon dilatation is performed on such patients because the high pressure of the balloon will lead to vascular endothelial injury and even the risk of vascular rupture, thus affecting the maturity of the internal fistula and even leading to surgical failure. The second is the multi-segment balloon dilatation to the elbow and closer to the heart of the blood vessels, often because the balloon guide wire is too deep, causing the patient’s vagal reflex, resulting in a sudden drop in heart rate, blood pressure, and blood oxygen, which is also unfavorable for surgical patients. Third, the operation of balloon dilatation is complicated and cumbersome, which is different from the simple operation of liquid dilatation, and requires experienced operators to operate. For the control of balloon pressure, it is necessary to have excellent technology. If the pressure is too small, the blood vessels cannot be dilated, and if the pressure is too large, the blood vessels will rupture. Fourth, brachial plexus anesthesia is required during balloon dilatation, and the anesthetic technique requirements of anesthesiologists are high. Finally, compared with liquid expansion and modified anastomosis, the patient’s intraoperative discomfort is more severe, and balloon expansion is expensive.

## 5. Conclusion

The results of this study show that balloon dilatation is more suitable for the establishment of internal fistula in patients with small blood vessels than liquid dilatation because multi-segment balloon dilatation has fewer contraindications and a wide range of trials and has a huge advantage in improving the blood flow, vascular diameter, maturity rate, and patency rate of small vascular internal fistula after operation, which is suitable for development. Therefore, we believe that balloon dilatation has a broad application prospect for the establishment of AVF in patients with small blood vessels compared with liquid dilatation, and in the long run, it is also a more economical choice, which can further benefit patients.

## Acknowledgments

The authors would like to thank all the reviewers who participated in the review and MJEditor (www.mjeditor.com) for their linguistic assistance during the preparation of this article.

## Author contributions

**Conceptualization:** Qiyu Kang, Yajie Hao, Huifeng Zhang, Weimin Yu, Xiaoguang Huang.

**Data curation:** Huifeng Zhang, Yajie Hao, Qiyu Kang, Weimin Yu, Xiaoguang Huang.

**Formal analysis:** Qiyu Kang, Yajie Hao, Xiaoguang Huang.

**Writing – original draft:** Huifeng Zhang, Yajie Hao, Qiyu Kang.

**Writing – review & editing:** Qiyu Kang, Yajie Hao, Weimin Yu, Xiaoguang Huang.

**Supervision:** Weimin Yu, Xiaoguang Huang.
